# A Retrospective Analysis of 190 Patients With Scoliosis Referred to a Private Australian Clinical Advisory Service Between 2017 and 2020

**DOI:** 10.1016/j.jcm.2024.08.003

**Published:** 2024-10-28

**Authors:** Sean Austin-Candler, Justine Carson, Robert Cheung, William Vuong, Alex Boakes, Roger M. Engel, Petra L. Graham, Jeb McAviney, Benjamin Thomas Brown

**Affiliations:** a Department of Chiropractic, Faculty of Medicine, Health and Human Sciences, Macquarie University, Sydney, New South Wales, Australia; b School of Mathematical and Physical Sciences, Faculty of Science and Engineering, Macquarie University, Sydney, New South Wales, Australia; c Sydney Scoliosis Clinic, Kogarah, New South Wales, Australia

**Keywords:** Scoliosis, Primary Health Care, Australia

## Abstract

**Objective:**

The aim of this study was to describe the demographic and clinical characteristics of patients with scoliosis from Australian primary care practices.

**Methods:**

A retrospective review of 190 patient records from August 2017 to April 2020 from a private Australian clinical advisory service database was performed. Deidentified demographic and clinical data were collated and analyzed, along with information regarding the referring practitioners and any accompanying clinical or paraclinical information. Numerical data were summarized with median and IQR, while categorical data were summarized with counts and percentages. Salient qualitative data from the advisory service records were also collated, coded, and summarized.

**Results:**

Patients were aged between 3 and 87 years; the majority (71%) of patients were female, with a median age of 16 years (IQR, 13; range, 3-87 years). The most common type of spinal deformity seen in the sample was scoliosis (92%), with hyperkyphosis (7%) and other deformity (1%) making up the remaining cases. There was a wide variety of scoliosis presentations; however, curves were commonly (45%) located in the thoracic region of the spine. Observed scoliosis cases were of moderate severity with a median Cobb angle measuring 26.5° (IQR, 20°). Reports of pain were in the lower trunk/pelvis (46%), the middle trunk (16%), or throughout multiple bodily regions (27%). Alterations in normal spinal anatomy (eg, hemivertebrae) were common (55% of cases). The majority (86%) of patient cases came from chiropractors, whereas 9% were from osteopaths, 4% from physiotherapists, and 1% from other types of practitioners (eg, medical practitioners).

**Conclusion:**

The findings from this study suggest that patients presenting to practitioners in primary care settings in Australia present with a range of scoliosis and related spinal deformity presentations.

## Introduction

Scoliosis is defined as a lateral deviation of the spine in the coronal plane measuring ≥10° (Cobb) with associated vertebral rotation.^1^ The condition may present at any point across the lifespan. Scoliosis is typically detected during dedicated screening programs or through patient/parent-initiated consultations with a primary care clinician (eg, a medical practitioner). Scoliosis is broadly classified as either idiopathic or nonidiopathic based on the presence or absence of observable/quantifiable etiologic factors. The prevalence and clinical features of scoliosis vary depending on etiology and are also influenced by sex, age, and the presence of comorbidities. The magnitude of spinal curvature in patients suspected of having scoliosis is measured using standing coronal plane x-rays. A diagnosis of scoliosis is confirmed if the observed lateral deviation measures ≥10° on a radiograph (Cobb).^2^

Politicians, policymakers, and health care professionals depend on epidemiological data to shape their decision-making processes, which is instrumental in crafting health care policies and programs,^3^ as well as guiding strategies for disease prevention, screening, diagnosis, and management. Therefore, it is imperative that information regarding the prevalence rates and burden of disease within a given population is reflective of the population's diversity. Additionally, these insights must be properly segmented to accommodate variations in disease manifestation and progression among different population subgroups.^4^ There is currently a paucity of data on the demographic and clinical characteristics of patients with scoliosis presenting to primary care settings in Australia. These data are important for discussions regarding the screening, diagnosis, and management of scoliosis in this region. Therefore, the aim of this study was to describe the demographic and clinical characteristics of Australian patients with scoliosis who presented to primary care practices.

## Methods

### Design

This study was a retrospective analysis of cross-sectional records from a private Australian clinical advisory service database.

### Data Source

Data were drawn from a clinical advisory service database from an Australian clinical network (ScoliCare) in Australia. The focus of this company is on the screening, diagnosis, and nonsurgical management of scoliosis and related spinal deformities. Any practitioner working in a primary care setting in Australia can upload clinical information and/or diagnostic imaging regarding patients with suspected scoliosis and/or related spinal deformities to a secure online portal for review or advice. A practitioner from the network provides the requestee with a detailed written report regarding potential diagnoses and management options for the patient. Management options may include comanagement and/or therapeutic services provided by ScoliCare.

In Australia, primary care is defined as: “The entry level to the health system and, as such, is usually a person's first encounter with the health system. It includes a broad range of activities and services, from health promotion and prevention, to treatment and management of acute and chronic conditions.”^5^ The first/primary contact a patient has with the health care system could be by a medical practitioner or an allied health practitioner such as a chiropractor or physiotherapist.

### Data Extraction

Deidentified demographic and clinical information from consecutive patient reports from August 1, 2017, to April 30, 2020, were collected along with information regarding the characteristics and geographic location (state or territory level data) of the referring practitioner. The types of patient-related clinical and/or paraclinical information (eg, radiographs) that accompanied the review request were also recorded. A list of the types of data that were extracted for this study can be found in the Supplemental File. All radiographic measurements were performed by a single practitioner (B.T.B.) using the Surgimap software (Nemaris Inc).

### Analysis

Numerical variables were summarized using median and IQR. Categorical variables were summarized using counts and percentages. All summary statistics were calculated using the R statistical program (version 4.1.2, R Core team).^6^ A free text description of abnormal spinal and/or pelvic anatomy was included with each record, where applicable. These qualitative data were collated, coded, analyzed, and summarized. NVivo software (version 12; QSR International Pty Ltd 2022) was used for the analysis of the qualitative data.

### Ethical Review

The project received ethical approval from the Macquarie University Human Research Ethics Committee (HREC number: 52020640714737).

## Results

There were 190 cases in this study. The majority (86%) of requests came from chiropractors, whereas 9% were from osteopaths, 4% from physiotherapists, and 1% from other types of practitioners (eg, medical practitioners). Almost half (49%) of the review requests came from practitioners in Victoria, 25% from New South Wales, 16% from Queensland, 4% each from South Australia and the Australian Capital Territory, and 2% from Western Australia. There were no review requests received from Tasmania or the Northern Territory during the review period.

The majority of practitioners (83%) used the service once; for those who used the service more than once, the median number of requests was 2 (IQR, 1). Referring practitioners provided diagnostic imaging with the review request in 98% of cases. The images were submitted in a variety of file formats (.JPEG, .PNG, or Digital Imaging and Communications in Medicine format (DICOM)). The imaging types included standard radiographs in 94% (175/187) of cases, and EOS imaging (Alphatec Holdings, Inc. Paris, France) in the remaining 6% (12/187) of cases. There was no high-level imaging (eg, computed tomography) provided by any of the referring practitioners. The median number of images provided by the practitioners was 4 (IQR, 3), which included both coronal and sagittal plane views in the majority (82%, 153/187) of cases. Either full spine views (45%, 84/187) or thoracic and lumbar views (43%, 81/187) were provided with most review requests. A radiological report was provided in 23% of cases, and clinical data in 88% of cases.

Of the 190 patient cases, 70% (133/190) were female, and the median age was 16 years (IQR, 13 years). The age range of patients was 3 to 87 years. More than half (58%) were of less than 18 years. The Risser classification of this group was Risser 0 (29%), Risser 1 (7%), Risser 2 (7%), Risser 3 (7%), Risser 4 (19%) and Risser 5 (4%). The triradiate cartilage, when visible, was unfused in 32% (25/77) of these patients. Pain was reported in 82% (107/130) of cases, and when explicitly described, was located in either the lower trunk/pelvis in 46% (46/99), the middle trunk in 16% (16/99), or throughout multiple bodily regions in 27% (27/99) of cases.

The primary deformity identified was scoliosis in 92% (175/190) of cases and hyperkyphosis in 7% (13/190) of cases. Other types of structural deformity (eg, queries regarding the diagnosis and management of leg length discrepancy) were 1% (2/190). The characteristics of the spinal deformity seen in this group are described in Table 1.

**Table 1:** Scoliosis and Related Spinal Deformity Characteristics

Deformity	Type, n (%)	Cobb Angle (°)	Range (°)	Sidedness, n (%)	Location, n (%)
Primary	Scoliosis, 175 (92.1) Hyperkyphosis, 13 (6.8)	Median, 26.5 (IQR, 20) Median, 70 (IQR, 17)	10-100 40-113	Left, 88 (50.9) Right, 85 (49.1) NA	Cervicothoracic, 1 (0.6) Thoracic, 78 (45.1) Thoracolumbar, 51 (29.5) Lumbar, 43 (24.9) Thoracic spine, 13 (100.0)
Secondary	Scoliosis, 111 (98.2) Hyperkyphosis, 2 (1.8)	Median, 21 (IQR, 16) Median, 66.5 (IQR, 12.5)	10-63 54-79	Left, 62 (55.9) Right, 49 (44.1) NA	Cervicothoracic, 2 (1.8) Thoracic, 68 (61.3) Thoracolumbar, 14 (12.6) Lumbar, 27 (24.3) Thoracic spine, 2 (100.0)
Tertiary	Scoliosis, 23 (92.0) Hyperkyphosis, 2 (8.0)	Median, 18 (IQR, 12) Median, 59.5 (IQR, 12.5)	10-38 47-72	Left, 12 (52.2) Right, 11 (47.8) NA	Cervicothoracic, 3 (13.0) Thoracic, 14 (60.9) Thoracolumbar, 2 (8.7) Lumbar, 4 (1.7) Thoracic spine, 2 (100.0)

In scenarios where there was more than 1 abnormal spinal curvature present, curves were classified based on magnitude (Cobb angle), with the curve demonstrating the largest deviation from normal physiological alignment classified as the primary curve. The “sidedness” of a curve was determined by the direction that the spine had deviated away from the midline on coronal plane images. The “location” of a spinal curvature was determined by the location (spinal region) of the apical vertebrae.

*%*, percentage (relative frequency); *n* = number (frequency); *NA*, not applicable.

Primary coronal plane spinal deformity was most common in the thoracic region (45%, 78/173). These cases ranged from minor curves (10°-20° [Cobb]) through to very severe curves (>56° [Cobb]), with median values in the moderate range (21°-35° [Cobb]). Median scoliosis Cobb angle values for the adult patients (>18 years) were higher than those seen in the pediatric group (30° [IQR, 22.8°] vs 24° [IQR, 17.0], respectively).

With respect to primary sagittal plane deformity, thoracic hyperkyphosis was the most common presentation. Curves ranged from subclinical (<40° [Cobb]) through to the surgical threshold (>80° [Cobb]) and beyond.^7^ The median hyperkyphosis angle was 70° (Cobb) (IQR, 17°). Median hyperkyphosis Cobb angle values for the adult patients (>18 years) were higher than those seen in the pediatric group (70° [IQR, 10.5°] vs 65.0° [IQR, 19.8°], respectively). There were 2 abnormal spinal curvatures (coronal and/or sagittal plane deformity) observed in 60% (115/190) of cases, and 3 abnormal spinal curvatures in 13% (25/190) of cases. Both secondary and tertiary deformities were also most common in the thoracic spine. There were 12% (12/99) cases of primary left-sided thoracic scoliosis in the pediatric portion of the sample.

As the images that accompanied the review requests came in several different file formats, were of varying quality, and often lacked reference markers of known sizes, it was not possible to accurately measure the exact magnitude of coronal or sagittal imbalance in these cases. Nor was it possible to adjust for projection, magnification, or stitching errors (combining 1 or more regional coronal, or sagittal plane spinal views into a single full spinal view) in the radiographic analyses. Furthermore, some parameters cannot be assessed from standard radiographic views, for example, leg length inequality or sacral obliquity. Instead, if the salient view was not available, the practitioner providing the review highlighted cases where there was evidence suggestive of a deviation from normal physiological alignment, for example, right coronal imbalance*.* Sagittal and coronal balance were only assessed if full spine views or stitched views demonstrating the seventh cervical vertebra down to the first sacral tubercle were available. Coronal imbalance was seen in 87% (125/143) of cases with the patient's trunk most often translated to the left in the coronal plane. Sagittal imbalance was seen in 66% (51/77) of cases with the patient's trunk most often translated posteriorly in the sagittal plane.

The sagittal spinal and pelvic parameters are described in Table 2. Leg length discrepancy was either confirmed or suspected in 69% (95/138) of cases, with the shorter leg being more common (63%) on the left (ie, left short leg on standing coronal plane views). Sacral obliquity (unlevelling of the sacral base in the coronal plane that is distinct from a leg length discrepancy) was either confirmed or suspected in 35.8% (47/131) of cases. Sacral obliquity was most often (82%) observed on the left (ie, low left sacral base on standing coronal plane views).

**Table 2:** Sagittal Plane Spinal and Pelvic Measurements

Parameter	n (%)	Angle (°)	Range (°)
Thoracic kyphosis^a^	88 (46)	Median, 41.5 (IQR, 22)	0-113
Lumbar lordosis^a^	125 (66)	Median, 47 (IQR, 19)	13-84
Sacral slope	126 (66)	Median, 39 (IQR, 11)	5-71
Pelvic tilt	92 (48)	Median, 11 (IQR, 11)	0-43
Pelvic incidence	92 (48)	Median, 48 (IQR, 14)	26-80

*%*, percentage (relative frequency); *n*, number (frequency).

a Cobb method.

Abnormal spinal and/or pelvic anatomy was seen in 55% (105/190) of cases. Confirmed or suspected sacral obliquity was the most common abnormality in the sample followed by different types of olisthesis, vertebral wedging (coronal and/or sagittal plane), spina bifida occulta, and transitional segments. Other forms of abnormality were seen less frequently (eg, hemivertebrae). A summary of the abnormal spinal and/or pelvic anatomy observed in the sample is described in Table 3.

**Table 3:** Summary of Abnormal Spinal and/or Pelvic Anatomy

Abnormality	Subtype	Number (%)
Sacral obliquity	NA	44 (23.3%)
Olisthesis	Anterolisthesis	10 (5.3%)
	Laterolisthesis	8 (4.2%)
	Retrolisthesis	4 (2.1%)
Vertebral wedging	Coronal plane	19 (10.0%)
	Sagittal plane	6 (3.2%)
Spina bifida	Cervical spine	1 (0.5%)
	Lumbar spine	4 (2.1%)
	Sacrum	8 (4.2%)
Transitional segment	Lumbarization	2 (1.1%)
	Sacralization	9 (4.8%)
Spondylosis	Cervical spine	1 (0.5%)
	Thoracic spine	3 (1.6%)
	Lumbar spine	5 (2.6%)
Posterior ponticle	Cervical spine	7 (3.7%)
Supernumerary ribs	Cervical spine	2 (1.1%)
Diffuse idiopathic skeletal hyperostosis	NA	2 (1.1%)
Facet tropism	Lumbar spine	1 (0.5%)
Hemivertebrae	Thoracic spine	1 (0.5%)
Hypoplastic ribs	Thoracic cage	1 (0.5%)

*%*, percentage (relative frequency); *NA*, not applicable.

## Discussion

The findings from this study describe the characteristics of patients with scoliosis being encountered by primary care practitioners in Australia. The prevalence of scoliosis observed in this sample is expected because they were derived from a clinical network that focuses on the treatment of scoliosis. Furthermore, scoliosis is more common than other types of spinal deformity in the general population. The female sex predilection in our sample is also a common finding in the majority of epidemiological studies of adult de novo degenerative scoliosis.^8^ In idiopathic scoliosis cases, smaller curves are distributed evenly between the sexes, but larger curves, similar to those seen in our sample, are more common in female patients.^9^ As there was limited clinical information provided with most review requests, it was not possible to confirm the diagnosis in the majority of cases. However, given the median age and clinical features (eg, curve type, location, and magnitude) of the sample, it is likely that patients with adolescent idiopathic scoliosis (AIS) were heavily represented in this sample. This could be interpreted in several ways. It could simply be the case that AIS presentations are common in chiropractic, osteopathic, and physiotherapy clinics in Australia. There is high-level evidence^10^ underpinning the treatment of AIS curves between 25° and 45° (Cobb) with thoracolumbosacral orthoses, and insufficient/limited evidence for other treatments that may typically be offered in primary care settings for this condition, for example, spinal manipulative therapy or general exercise.^11^

The median Cobb angle of the scoliosis cases seen in the sample falls within the recommended range for bracing treatment in skeletally immature patients,^12^ but also aligns closely with findings from cross-sectional studies from other regions where scoliosis screening is not routinely performed.^13^^,^^14^ This could be interpreted as practitioners making appropriate and timely referrals to relevant care providers, or alternatively, that there are a high number of moderate scoliosis cases being seen in primary care settings. The latter is unlikely, given that most epidemiological studies^15^, ^16^, ^17^, ^18^, ^19^, ^20^ on AIS from around the world demonstrate that the majority of curves are smaller in magnitude (<20° [Cobb]) than those seen in our sample. An alternate explanation is that primary care practitioners may be referring patients who have been unresponsive to other forms of conservative care.

A large proportion (82%) of the sample reported pain. This pain, when explicitly described, was most commonly located in the lower trunk/pelvis or middle trunk. This finding is not unexpected given that bodily pain is a common finding across the lifespan in both treated (surgical and nonsurgical) and untreated patients with scoliosis compared to controls.^21^, ^22^, ^23^, ^24^

A retrospective analysis of radiographic reports from patients attending a student outpatient health center in New Zealand revealed that osseous and pathologic spine anomalies were seen in 68% of patients.^25^ There are differences in the prevalence and type of spinal anomalies seen in our study. This may be influenced by sample size or the unique clinical population in our study.

A primary left-sided thoracic scoliosis in a pediatric patient (≤18 years) may be suggestive of a nonidiopathic curve, as there is an association between these types of presentations and abnormalities of the brain and/or spinal cord (eg, syringomyelia).^26^ Davids et al^26^ conducted a systematic literature review and meta-analysis of 18 studies involving 4746 patients with AIS who had received preoperative magnetic resonance imaging. The authors reported an 8% (95% CI, 6%-12%) pooled prevalence of neuroaxis abnormalities. There was a 12% prevalence of primary left-sided thoracic curves in the pediatric subset of our sample. A left-sided thoracic curvature is not pathognomonic with neuroaxis pathology^27^ but does warrant further investigation as there may be a specific (nonidiopathic) cause of the patient's scoliosis.

### Limitations and Future Studies

It must be acknowledged that this is a small sample and the data have been drawn from a private clinical advisory service database, and therefore cannot be used to comment directly on the population prevalence statistics of scoliosis and related spinal deformity in this region. Such metrics could only be obtained from large epidemiological studies such as from records from school screening programs. Given that the clinical network providing the review service also offered bracing treatment, which is not typical in primary care settings in Australia, referrals may have also been influenced by the types of services offered. Furthermore, any educational and promotional content produced by the clinical network would have also influenced patient referrals in a similar manner.

In the future, it would be of benefit to improve upon the granularity of the data that is captured by the clinical advisory service. This would allow researchers to explore cross-sectional associations between salient independent variables and different scoliosis characteristics.

Data were missing for some variables due to the variation in the quality and type of the images provided. A single practitioner (B.T.B.) with additional training in scoliosis and related spinal deformity performed all the radiographic measurements digitally. This method is comparable to manual measurement techniques and demonstrates good to excellent interrater reliability.^28^^,^^29^ Moreover, the experience level of an individual does not appear to affect the interreliability and intrareliability of the measurement in practitioners who have been trained in the digital measurement of Cobb angles.^30^ However, it can be assumed that there is a small degree of error in the radiographic measures provided.

Based on the observed versus expected practitioner numbers for each state and territory, there is an overrepresentation of referrals from Victoria. Furthermore, there is a lack of referrals from both medical practitioners and physiotherapists. The network providing the clinical advisory service offers continuing professional development seminars/modules. Attendees at these events are predominantly members of the chiropractic and osteopathic professions which may explain the observed imbalance in the referring professions represented.

## Conclusion

The findings from this study show that there was a range of scoliosis and related spinal deformity presentations being seen by practitioners in primary care settings in Australia. The majority of patients were females, in their mid-late teens, with moderate scoliosis in the thoracic region of the spine. These data provide a preliminary view of the demographics and characteristics of scoliosis and spinal deformity patients in Australia. In the absence of epidemiological studies or data from school screening programs, our results may inform preliminary discussions regarding the screening, diagnosis, and management of scoliosis in this region.

## Appendix Supplementary materials

Image, application 1

## Footnotes

Supplementary material associated with this article can be found in the online version at doi:10.1016/j.jcm.2024.08.003.
