# Supplementary material for: A Retrospective Analysis of 190 Patients With Scoliosis Referred to a Private Australian Clinical Advisory Service Between 2017 and 2020
Source: J Chiropr Med. 2024 Oct 28;23(4):171–7. doi: 10.1016/j.jcm.2024.08.003 (PMC11701887; doi:10.1016/j.jcm.2024.08.003)
Supplement: Supplementary file 1 [file mmc1.docx]

**Supplementary File 1: Types of data collected**

| **Practitioner Level Data** | **Data** | **Explanation** |
| --- | --- | --- |
|  | Provider Type | Type of referring practitioner who requested the review |
|  | Number of requests | The number of review requests made by the referring practitioner during the review period |
|  | Geographic location | The state or territory in which the referring practitioner was located |
|  | Imaging | Type, region and number of views of any imaging data provided by the referring practitioner |
|  | Report | Whether a diagnostic imaging report was provided by the referring practitioner |
| **Patient Level**  **Data** | Age | The age (years) of the patient at the time of the review request |
|  | Sex | Sex of the patient |
|  | Pain | Whether the patient reported pain, and if so, the bodily location of the pain |
| **Imaging Data** | Primary deformity | The type (scoliosis or hyper-kyphosis), magnitude (Cobb angle), location (spinal region) and sidedness (where applicable) of the most prominent/largest spinal deformity on diagnostic imaging |
|  | Secondary deformity | The type (scoliosis or hyper-kyphosis), magnitude (Cobb angle), location (spinal region) and sidedness (where applicable) of the second most prominent/largest spinal deformity on diagnostic imaging |
|  | Tertiary deformity | The type (scoliosis or hyper-kyphosis), magnitude (Cobb angle), location (spinal region) and sidedness (where applicable) of the third most prominent/largest spinal deformity on diagnostic imaging |
|  | Sagittal Plane Measurements | Measurements and classifications of the sagittal plane spinal and pelvic parameters on diagnostic imaging (includes sagittal imbalance, sacral slope, pelvic incidence and pelvic tilt) |
|  | Coronal plane measurements | Measurements and classifications of the coronal plane spinal parameters on diagnostic imaging (includes coronal imbalance) |
|  | Markers of skeletal maturity | Degree of iliac crest ossification/fusion (Risser classification) or visualisation of the tri-radiate cartilage on diagnostic imaging. The Risser classification system is used to grade skeletal maturity based on the level of ossification and fusion of the iliac crest apophyses (Grades I-V) |
|  | Potentially ominous presentations | A primary left-sided thoracic scoliosis in a paediatric patient (≤18 years) is considered a sign for clinicians to be wary of of as there is an association between these types of presentations and abnormalities of the brain and/or spinal cord *e.g.* syringomyelia. |
|  | Leg length discrepancy | Evidence of a difference in the length of the paired limbs on diagnostic imaging |
|  | Abnormal spinal/pelvic anatomy | Evidence of a spinal anomaly that is observed in addition to the primary, secondary or tertiary deformity on diagnostic imaging |
